# Multiple CAR-T cell therapy for acute B-cell lymphoblastic leukemia after hematopoietic stem cell transplantation: A case report

**DOI:** 10.3389/fimmu.2022.1039929

**Published:** 2022-11-17

**Authors:** Lei Deng, Yu Xiaolin, Qian Wu, Xiaochen Song, Wenjun Li, Yixi Hou, Yue Liu, Jing Wang, Jun Tian, Xiaona Zuo, Fang Zhou

**Affiliations:** ^1^ Hematology Department, The 960th Hospital of The People's Liberation Army (PLA) Joint Logistics Support Force, Jinan, China; ^2^ Nuclear Medicine Department, The 960th Hospital of the People’s Liberation Army (PLA) Joint Logistics Support Force, Jinan, China; ^3^ Department of Pathology, Beijing Boren Hospital, Beijing, China

**Keywords:** B-ALL, extramedullary recurrence, hematopoietic (stem) cell transplantation (HCST), cytosine, CAR (chimeric antigen receptor) T cells

## Abstract

B-cell acute lymphoblastic leukemia (B-ALL) is the most common childhood malignancy. The cure rate has reached 90% after conventional chemotherapy and hematopoietic stem cell transplantation (HSCT), but the prognosis of patients with relapsed and refractory (R/R) leukemia is still poor after conventional treatment. Since FDA approved CD19 CAR-T cell (Kymriah) for the treatment of R/R B-ALL, increasing studies have been conducted on CAR-T cells for R/R ALL. Herein, we report the treatment of a patient with ALL who relapsed after allogeneic HSCT, had a complete remission (CR) to murine scFv CD19 CAR-T but relapsed 15 months later. Partial response was achieved after humanized CD19 CAR-T treatment, and the patient finally achieved disease-free survival after sequential CD22 CAR-T treatment. By comparing the treatment results of different CAR-T cells in the same patient, this case suggests that multiple CAR-T therapies are effective and safe in intramedullary and extramedullary recurrence in the same patient, and the expansion of CAR-T cells and the release of inflammatory cytokines are positively correlated with their efficacy. However, further clinical studies with large sample sizes are still needed for further clarification.

## Introduction

B-cell acute lymphoblastic leukemia (ALL) is the most common childhood malignancy and is usually treated with chemotherapy and allogeneic hematopoietic stem cell transplantation (1). The cure rate has reached 90%, but the prognosis of patients with relapsed and refractory (R/R) leukemia after conventional treatment is very poor (2, 3). Patients with recurrence after allogeneic hematopoietic stem cell transplantation (allo-HSCT) are usually treated with donor lymphocyte infusions (DLI) (4, 5). DLI can induce complete remission (CR); however, many patients do not achieve sustained CR (6, 7).

Drugs such as monoclonal antibodies (anti-CD20), anti-CD19 bi-specific T cell binding agents, and anti-CD22 antibody-drug conjugates have shown unexpected results both in the prophase and R/R settings and continue to change the treatment paradigm for ALL (8–10). In one phase 3 trial (11), inotuzumab ozogamicin, an anti-CD22 antibody conjugated to caricomycin, showed significantly higher response rates and better progression-free survival (PFS) than standard intensive chemotherapy in adults with R/R B-ALL. In addition, more patients became minimal residual disease (MRD)-negative and required allo-HSCT. In another randomized phase 3 trial involving adults with Ph-negative R/R B-cell precursor ALL (12), treatment with blinatumomab, a bi-specific monoclonal antibody construct that enables CD3-positive T cells to recognize and eliminate CD19-positive ALL blasts, resulted in significantly longer overall survival (OS) than standard chemotherapy. The blinatumomab group also had a 29% lower risk of death than the chemotherapy group.

Since the first CD19 chimeric antigen receptor T (CAR-T) cell (Kymriah) was approved by the FDA for R/R acute lymphoblastic leukemia, several CD19 CAR-T cells have been approved by the FDA, including Yescarta, Tecartus, and Breyanzi (13). Kymriah and other CD19 CAR-Ts have also shown high CR rates (70%-93%) in r/r B-ALL patients (14–19). However, some patients did not respond, and some relapsed within one year (43-55%) (15–19). However, one study showed that CD22 CAR-T cell therapy had a 74% response rate in 21 patients with R/R B-ALL (20). CD22 CAR-T cell therapy has a good response rate, even in patients who failed to respond to CD19 CAR-T therapy or those who have relapsed. Herein, we report the treatment of a patient who relapsed after allo-HSCT, relapsed after murine scFv CD19 CAR-T therapy, had a partial response after humanized CD19 CAR-T therapy and finally achieved disease-free survival after sequential CD22 CAR-T therapy.

## Case

**Figure 1:**
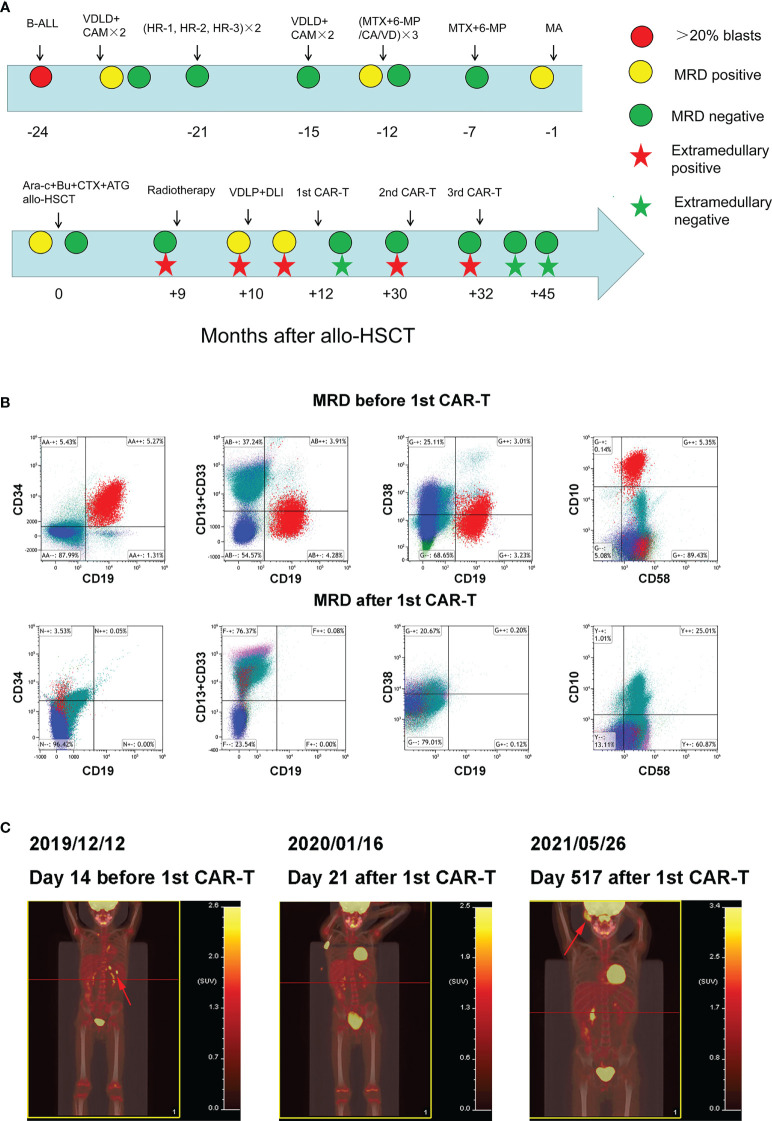
Clinical treatment process and the response of the patient **(A)** Clinical treatment process and response of the patient. **(B)** Bone marrow MRD before and after 1^st^ CAR-T treatment. **(C)** Whole body PET/CT before and after 1^st^ CAR-T treatment.

On November 5, 2016, a five-year-old male patient was admitted to the hematology department of a local hospital due to a neck mass. Physical examination showed scattered ecchymosis on the skin, the lymph nodes were swollen in the neck, axilla, and groin, and a mass of approximately 10 cm × 8 cm × 2 cm was detected on the right side of the neck with poor mobility, no tenderness, no congestion in the pharynx, grade 2 tonsil enlargement, and no abnormalities in the cardiopulmonary region, abdomen, or nervous system. Results of a routine blood examination revealed a white blood cell (WBC) count of 16.66×10^9^/L, hemoglobin (HB) 114 g/L, and platelet (PLT) count of 172×10^9^/L. Bone marrow cytology showed that hyperplasia was active, the proportion of granulocytes was low, the proportion of lymphocytes, mainly primitive naive lymphocytes, had increased (83.5%), and the proportion of peripheral blood blast cells was 59%. Immunophenotyping showed that abnormal cells accounted for 84.66% of nuclear cells and expressed CD34, human leukocyte antigen DR(HLA-DR), CD123, CD10, CD19, cCD79a, terminal deoxynucleotidyl transferase (TDT), CD38, and CD22. Cytosolic immunoglobulin M (CIgM), secretory immunoglobulin M (sIgM), CD117, CD20, CD7, CD33, CD15, CD13, CD11b, CD64, CD36, CD4, CD14, CD56, myeloperoxidase (MPO), cytoplasmic CD3 (cCD3), and membrane CD3 (mCD3) were not expressed. All 43 fusion genes were negative. The results of the karyotype analysis were as follows: 58-60, XY, + 4, + 5, + 6, + 7, + 14, + 17, + 18, and + 22 [CP5]. A diagnosis of acute lymphoblastic leukemia was made. The course of treatment for this patient is shown in **Figure 1A**.

On November 10, 2016, the VDLD (Vincristine, Daunorubicin, L-asparaginase, and Dexamethasone) chemotherapy regimen was administered. On day 15, bone marrow cell morphology showed a severe reduction of bone marrow hyperplasia and a naive lymphocyte proportion of 12%. On day 33, marrow cell morphology revealed an MRD of 4.61%. The MRD was negative after two courses of CAM (Cyclophosphamide, Cytarabine, and Azathioprine) regimen consolidation. Two rounds of chemotherapy with the HR-1 (Dexamethasone, Vincristine, High dose methotrexate, Cyclophosphamide, Cytarabine, and L-asparaginase), HR-2 (Dexamethasone, Vindesine, High dose methotrexate, Ifosfamide, Vincristine, and L-asparaginase), and HR-3 (Dexamethasone, High dose cytarabine, Etoposide, and L-asparaginase) protocols began in February 2017, and the VDLD+CAM×2 regimen began on August 27, 2017. On November 8, 2017, the MTX+6-MP/CA/VD (Methotrexate, Azathioprine, Cyclophosphamide, Cytarabine, Vincristine, and Dexamethasone) regimen was administered, during which time the bone marrow MRD was 0.18%. The second cycle of MTX+6-MP/CA/VD chemotherapy was initiated in December 2017 and the third cycle of MTX+6-MP/CA/VD chemotherapy was initiated in March 2018. Subsequently, the MTX+6-MP/CA/VD regimen was used for maintenance treatment. During the period, multiple lumbar punctures were performed and chemotherapeutic drugs were injected to prevent central nervous system leukemia, and cerebrospinal fluid examination showed no abnormalities.

On September 27, 2018, the patient visited our hospital for further diagnosis and treatment, and a physical examination revealed no abnormalities. Results of a routine blood examination were: WBC count, 2.77×10^9^/L; HB 89 g/L; and PLT 353×10^9^/L. We also noted bone marrow cytological remission, MRD of 1.1%, and CD9, CD10, CD19, CD20, CD34, CD38, and CD58. Chromosome analysis results were 46, XY [20]. MA (Methotrexate and Cytarabine) chemotherapy was subsequently administered. On November 5, 2018, bone marrow cytology revealed that the MRD was 2.01%. On November 21, 2018, the pretreatment regimen of BUCY+ARA-C+ATG (Busulfan, Cyclophosphamide, Cytarabine, and Antithymocyte globulin) was started. Allo-HSCT (MNC 11.8×10^8^/kg, CD34+ cells 9.84×10^6^/kg) was performed on November 28 and 29, and the donor was the father of the patient with 5/10 HLA matches. Multiple bone marrow test results after transplantation were negative for MRD.

In September 2019 (the ninth month after transplantation), the patient experienced left scrotal swelling and pain; the bone marrow morphology was relieved, MRD was negative, and donor chimerism was 98.89%. A biopsy of the mass (left bolus biopsy tissue) showed that it was consistent with B-cell lymphoblastic leukemia or lymphoma. Immunohistochemistry results were: CD10 (+), the TdT (+), MPO (+), CD43 (+), CD79a (+), aired box gene 5+ (Pax-5 +), leucocyte common antigen (LCA) (focal +), CD20 + (part), P53 (approximately + 25%), CD3 (in +), CD2 (in +), CD21 (–), the Placental alkaline phosphatase (PLAP) (-), Mum - 1 (-), Bcl-6 (-), CD30 (-), CD5 (-), and Ki-67 (approximately 50% +). *In situ* hybridization results: Epstein-Barr virus-encoded small RNA (EBER) (-). Three-dimensional conformal radiotherapy (200 cGy/time × 13 times) was administered to the left testis and the left scrotum was significantly reduced. Bone marrow morphology on October 26 revealed: 1.55% naive lymphocytes, 0.78% MRD, and 99.78% donor chimerism. VDLD chemotherapy was then administered. On the 14th day of chemotherapy, bone marrow morphology showed low proliferation, and the percentage of proto-juvenile cells was 5%. Donor lymphocyte infusion (MNC 1.29×10^8/^kg) was performed. Bone marrow morphology 12 days after infusion (on December 2) revealed hyperplasia was active, and that the proportion of primary and juvenile lymphocytes was 3.5%. MRD was 5.35%, and this population of cells expressed CD10, CD19, CD34, and CD58 (**Figure 1B**). The donor chimerism rate was 93.47%. PET/CT revealed: multiple lymph nodes with increased FDG metabolism in the left side of the abdominal aorta, posterior to the pancreas, and splenic hilum, considering leukemia infiltration; and after radiotherapy, the left testicle was enlarged compared to the contralateral testicle, and FDG metabolism was not significantly increased (**Figure 1C**).

On December 16, 2019, peripheral blood mononuclear cells were collected from the patient and murine scFv CAR-T cells were cultured at Shanghai YaKe Biotechnology Ltd. After pretreatment with the FC (Fludarabine, cyclophosphamide) regimen, CAR-T cells were infused on December 26. On day seven after infusion, the patient showed increased heart rate, elevated transaminase levels, and elevated inflammatory factors, and the CRS was assessed as grade 1 (**Table 1**). The CRS classification scheme is based on the report of Lee DW et al. (21). On January 14, 2020, the bone marrow morphology was relieved, and immunoreactivity was negative. PET/CT revealed that: the left para-abdominal aorta, posterior pancreas, and splenic hilar lymph nodes were slightly smaller than the anterior ones, the FDG metabolism was not significantly increased, and FDG metabolism did not increase after radiotherapy for left testicular infiltration (**Figure 1C**). These results suggested that the patient was in CR, which persisted for 15 months after discharge.

**Table 1:** Characterization of the three infusions of CAR-T cells.

	1st CAR-T	2nd CAR-T	3rd CAR-T
Vector	lentivirus	lentivirus	lentivirus
scfv	Murine	Humanized	Humanized
CAR structure	Anti-CD19(FMC63)scFV-CD8a-4-1BB-CD3ζ	Anti-CD19 scFV-CD8a-4-1BB-CD3ζ	Anti-CD22 scFV-CD8a-4-1BB-CD3ζ
Derived	Autologous	Allogenic	Allogenic
Total cells dose	1.85×10^8^	2.4×10^8^	2.81×10^8^
CAR-T ratio	79%	69.53%	56.4%
Weight (kg )	28	31	31
CAR-T cells (/kg )	5.2×10^6^	5.3×10^6^	5.17×10^6^
Blasts% pre-CAR-T cell infusion	EM+ and 5.35% in BM	EM+ and MRD-	EM+ and MRD-
ECOG score pre-CAR-T cell infusion	1	1	1
Expression of CD19/CD22 Before treatment	CD19+CD22+	CD19+CD22+	CD19+CD22+
Maximum of CAR-T cells/CD3+ cells	0.19%	3.3%	17.4%
Precondition	FC	FC	FC
Response	CR	Mass decrease	CR
CRS	1	1	2

CAR, chimeric antigen receptor; scFv, single-chain fragment variable; ECOG, Eastern Cooperative Oncology Group; EM, extramedullary; BM, bone marrow; MRD, minimal residual disease; FC, fludarabine cyclophosphamide; CR, complete remission; CRS, cytokine release syndrome.

**Figure 2:**
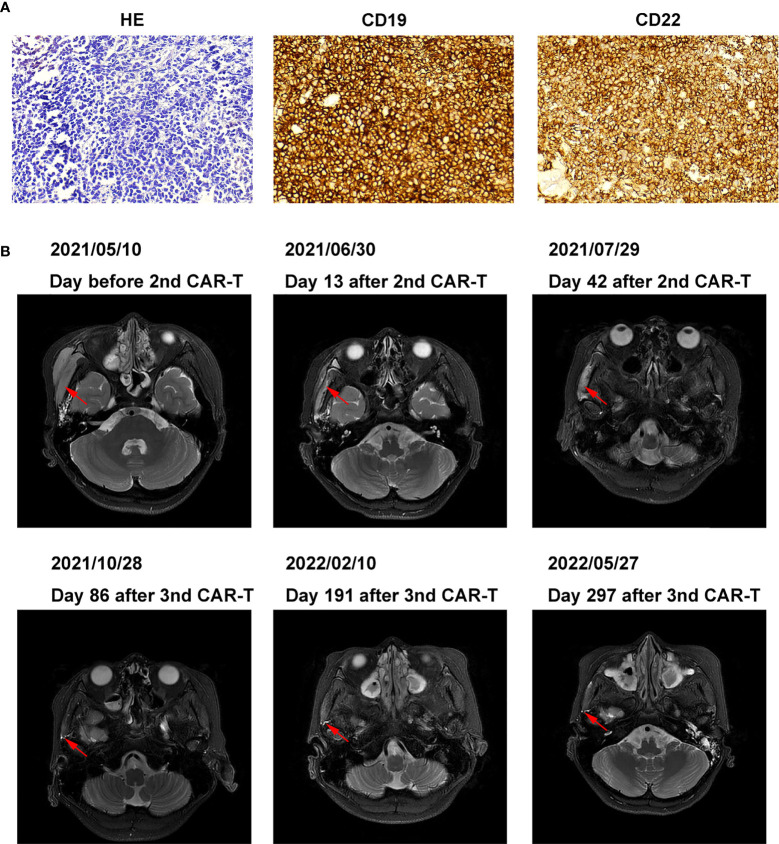
Pathology, histochemistry, and MRI images of right facial mass **(A)** Pathology and histochemistry of right facial mass. **(B)** MRI images of the right facial mass before and after 2nd, the 3rd CAR-T cell treatment.

On April 11, 2021, the patient developed a facial mass on the right side that gradually increased in size. On May 6, an ultrasound examination revealed a 44 mm × 8 mm mass with a poorly defined border and an irregular shape. A biopsy under B-ultrasound guidance was performed on May 13. Pathological findings of the biopsy tissue of the right facial mass showed a lymphoproliferative lesion, consistent with B-lymphoblastic leukemia/lymphoma (**Figure 2A**). Immunohistochemical staining results were: CD79a+, CD43+, TdT+, CD20 scattered+, PAX-5 scattered+, P53 approximately 25% weak+, CD5-, CD23-, CD3-, CD2-, CD7-, MPO-, and Ki-67 (approximately 60%+). *In situ* hybridization results: FBER. On May 22, examinations showed bone marrow morphologic remission and negative MRD, with complete donor-type chimerism. PET/CT revealed: newly found space-occupying lesions with increased FDG metabolism in the subcutaneous soft tissue of the right face; and newly observed increased FDG metabolism in the nasopharynx and slightly increased bilateral small cervical lymph nodes (**Figure 1C**). On June 8, peripheral blood mononuclear cells were collected from the donor (the patient’s father), and humanized CD19 CAR-T cells were cultured. After pretreatment with the FC regimen, CAR-T cells were injected on June 17. On the fifth day after infusion, the patient developed a low fever (37.5°C) without other discomfort symptoms. CRS was rated grade 1. Facial MRI on days 13 and 42 after infusion revealed that the mass was smaller than before infusion (**Figure 2B**).

**Figure 3:**
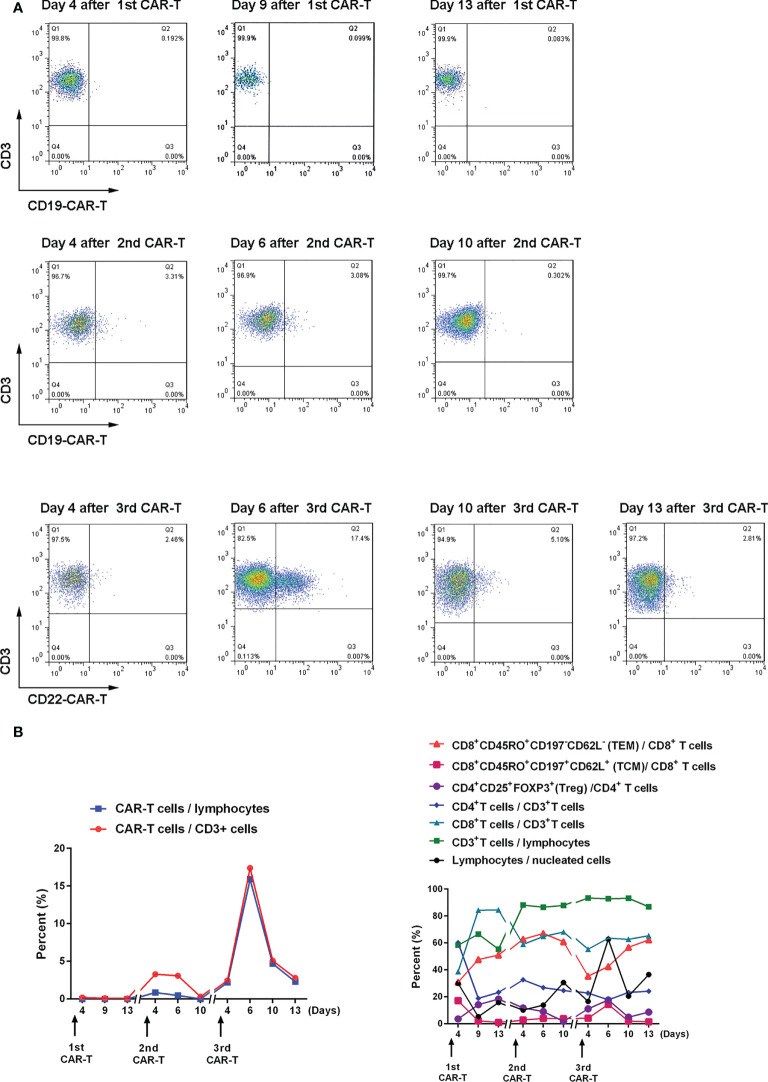
Expansion and changes of T cell subsets after CAR-T cell therapy **(A)** The proportion of CAR-T cells in CD3^+^ cells after CAR-T cell therapy was detected by flow cytometry. **(B)** Changes in the proportion of T cell subsets after CAR-T cell therapy. The memory phenotype is in bulk T cells.

On July 30, 2021, the patient was pretreated with the FC protocol, and on August 3, humanized CD22 CAR-T cells cultured from the peripheral blood mononuclear cells of the donor (the patient’s father) were transfused back. On the first day after the infusion, the patient developed a fever with the highest temperature of 38.6°C. Subsequently, the patient continued to have repeated high fevers, with the highest temperature of 39°C, and a cough accompanied by chest tightness and breathlessness. Chest CT showed a pulmonary infection, which improved after antipyretic symptomatic treatment and anti-infective treatment. Drugs used included ibuprofen, meropenem, voriconazole, and caspofungin. No steroid hormones were used. CRS was graded as grade 2. As shown in **Figure 3**, CAR-T cell expansion and cytokine elevation were the most dramatic after the third CAR-T cell infusion (**Supplementary Figure 1**). After the third CAR-T cell infusion, the tumor was significantly reduced in size, with no activity in the lesion 191 and 297 days (February 10, 2022, and May 27, 2022) after the third CAR-T cell infusion (**Figure 2B**).

## Discuss

In this patient with acute lymphoblastic leukemia, the first extramedullary (testicular) recurrence occurred more than nine months after allogeneic transplantation. After radiotherapy, the extramedullary lesions disappeared, but the bone marrow MRD was positive. The MRD increased after chemotherapy and donor lymphocyte infusion. After the first CAR-T cell treatment, the MRD was negative. Extramedullary (facial) recurrence occurred after 15 months, which improved after two CAR-T cell treatments, following which the patient continued to live disease-free.

Occasionally (≤ 2%), testicular recurrence occurs in acute lymphoblastic leukemia (22). Ding et al. (23) showed that testicular recurrence might directly evolve from leukemia clones that survive chemotherapy. It is also likely to have relapsed independently from the bone marrow. Radiotherapy is a good choice for patients with isolated testicular recurrences (24). Besides, CAR-T therapy for testicular recurrence has been started in several studies. In the study of Chen X et al. (25), all 7 patients had CR. One patient had bone marrow recurrence 6 months after CAR-T treatment, and 6 patients were still in remission during the follow-up period (median 14 months). Only 5 patients developed grade 1 CRS, and the remaining two patients did not develop CRS. Rubinstein JD et al. (26) and Yu J et al. (27) each reported 1 case of ALL with testicular recurrence and remission after CAR-T treatment.

Based on flow cytometric assessment of CD19 expression in B-ALL, relapse after CD19 CAR-T cell treatment can be divided into two groups: CD19-negative relapse and CD19-positive relapse (28, 29). When the patient relapsed after the first CD19 CAR-T cell treatment, immunohistochemistry showed strong CD19 positivity (**Figure 2A**), and the patient was classified as a CD19-positive relapse. Positive relapse is usually due to low potency or CAR-T cell loss. Several factors limit CAR-T cell power and efficacy, including limited long-term persistence, immunosuppressive tumor microenvironment, and intrinsic dysfunction associated with T cell exhaustion (30–33).

In some cases of treatment failure, secondary infusions of CD19 CAR-T cells were not reproducibly successful if the CD19 expression in leukemic cells remained high, possibly partly due to immune-mediated clearance of murine scFv CAR-T cells (34–36). Previous studies have shown that humanized CAR-T remains effective in patients who relapsed after murine scFv CAR-T cells treatment (37, 38). Therefore, we treated the patient with a second humanized CD19 CAR-T, resulting in a reduction of the patient’s lesions. In this patient, the expression rate of CD19 was still very high at the time of recurrence (**Figure 2A**). Among patients with CD19-positive ALL who relapsed after murine scFv CD19 CAR-T treatment, the humanized CD19 CAR-T CR rate (50%-64%) was still lower than in patients who had not received CAR-T therapy (37, 38). We believe that it may be caused by the resistance of tumor cells to CD19 CAR-T, and the mechanism needs to be further studied. In contrast, approximately 80% of patients with ALL after CD19 CAR-T cell therapy had CR after CD22 CAR-T cell therapy, which was not different from patients who did not receive CD19 CAR-T cell therapy (20, 39). Although we did not detect the mean fluorescence intensity (MFI) of CD19 and CD22, CD19 and CD22 in **Figure 2A** were analyzed by Image-Pro Plus software through the method of Liu X et al. (40). The mean density of CD19 (0.32 ± 0.01) was higher than that of CD22 (0.21 ± 0.00), indicating that the expression intensity of CD19 was still very high. The success of CD22 CAR-T may be due to the resistance of tumor cells to CD19 CAR-T at this time, but not to CD22 CAR-T.

The third CAR-T cell therapy yielded surprising results, with the patient’s lesions continuing to shrink with no significant activity. We also found a positive correlation between CAR-T cell expansion and the efficacy of the second and third CAR-T therapy in the same patient. The inflammatory cytokines IFNγ, SIL-2R, TNFα, and IL-6 also showed a positive correlation with CAR-T efficacy (**Supplementary Figure 1**). Treg cells inhibit excessive immune responses by expressing CTLA4 and secreting IL-10 and TGFβ (41), reflected in changes to Treg and IL-6 levels along with changes in inflammatory cytokines.

Relapse is observed in 30-60% of patients with acute lymphoblastic leukemia after CD19 CAR-T cell therapy, mostly within one year (16, 19, 28, 42, 43). Data from both murine studies and integration site analysis after adoptive T cell transfer in humans suggest that long-term persisting T cells are predominantly derived from stem cell-like memory T cells (TSCM) and central memory T cell (TCM) compartments of the infused product (44). In this patient, effective memory T Cell (TEM) increased after three CAR-T cell treatments and only began to decrease six days after the second CAR-T cell treatment. TCM increased significantly after the first and third treatments (17.1% and 14.4%, respectively) and increased to 4% after the second treatment (**Figure 2B**). Our data suggest that this is also likely to be true after CAR-T cell therapy.

In some studies, CAR-T therapies result in antigen escape/loss, and the rate of CD19-negative recurrence in ALL patients is 7%-25% (14–16, 34, 45, 46), while more patients have CD19-positive recurrence. To combat immune escape, studies continue to combine CAR T cells with radiation (47), checkpoint suppression (48), vaccines (49), or other immune agonists (50, 51). Another approach is to simultaneously target more than one antigen on cancer cells, such as CD19, CD20, and CD22 (20, 52–54). We believe that even if patient antigen escapes/loss, other antigens can still be searched, and more studies are still in progress. Summers et al. (55) showed no benefit of a second allogeneic HSCT after CAR-T treatment for patients with recurrence after HSCT. Moreover, the outcome of the second HSCT is usually worse in B-ALL patients (56–58). This patient is currently in sustained remission 14 months after the third CAR-T treatment, and we will continue to follow him closely.

By comparing the results of different CAR-T cells in the same patient, this case suggests that multiple CAR-T therapies is effective and safe in both intramedullary and extramedullary recurrence in the same patient and that CAR-T cell expansion and inflammatory cytokine release are positively associated with its efficacy. Further clinical studies with large sample sizes are needed for further clarification.

## Data availability statement

The original contributions presented in the study are included in the article/**Supplementary Material**. Further inquiries can be directed to the corresponding author.

## Ethics statement

The studies involving human participants were reviewed and approved by The Ethical Committee of the 960th Hospital of the People’s Liberation Army. Written informed consent to participate in this study was provided by the participants’ legal guardian/next of kin.

## Author contributions

LD and FZ contributed to data collection, data analyses, wrote the draft of the paper and had final responsibility to submit for publication. QW, XY, QW, XS, WL, and YH contributed to clinical protocol. All authors contributed to the article and approved the submitted version.

## Conflict of interest

The authors declare that the research was conducted in the absence of any commercial or financial relationships that could be construed as a potential conflict of interest.

## Publisher’s note

All claims expressed in this article are solely those of the authors and do not necessarily represent those of their affiliated organizations, or those of the publisher, the editors and the reviewers. Any product that may be evaluated in this article, or claim that may be made by its manufacturer, is not guaranteed or endorsed by the publisher.
