# Supplementary material for: Multiple CAR-T cell therapy for acute B-cell lymphoblastic leukemia after hematopoietic stem cell transplantation: A case report
Source: Front Immunol. 2022 Nov 17;13:1039929. doi: 10.3389/fimmu.2022.1039929 (PMC9713842; doi:10.3389/fimmu.2022.1039929)
Supplement: Supplementary file 1 [file DataSheet_1.doc]

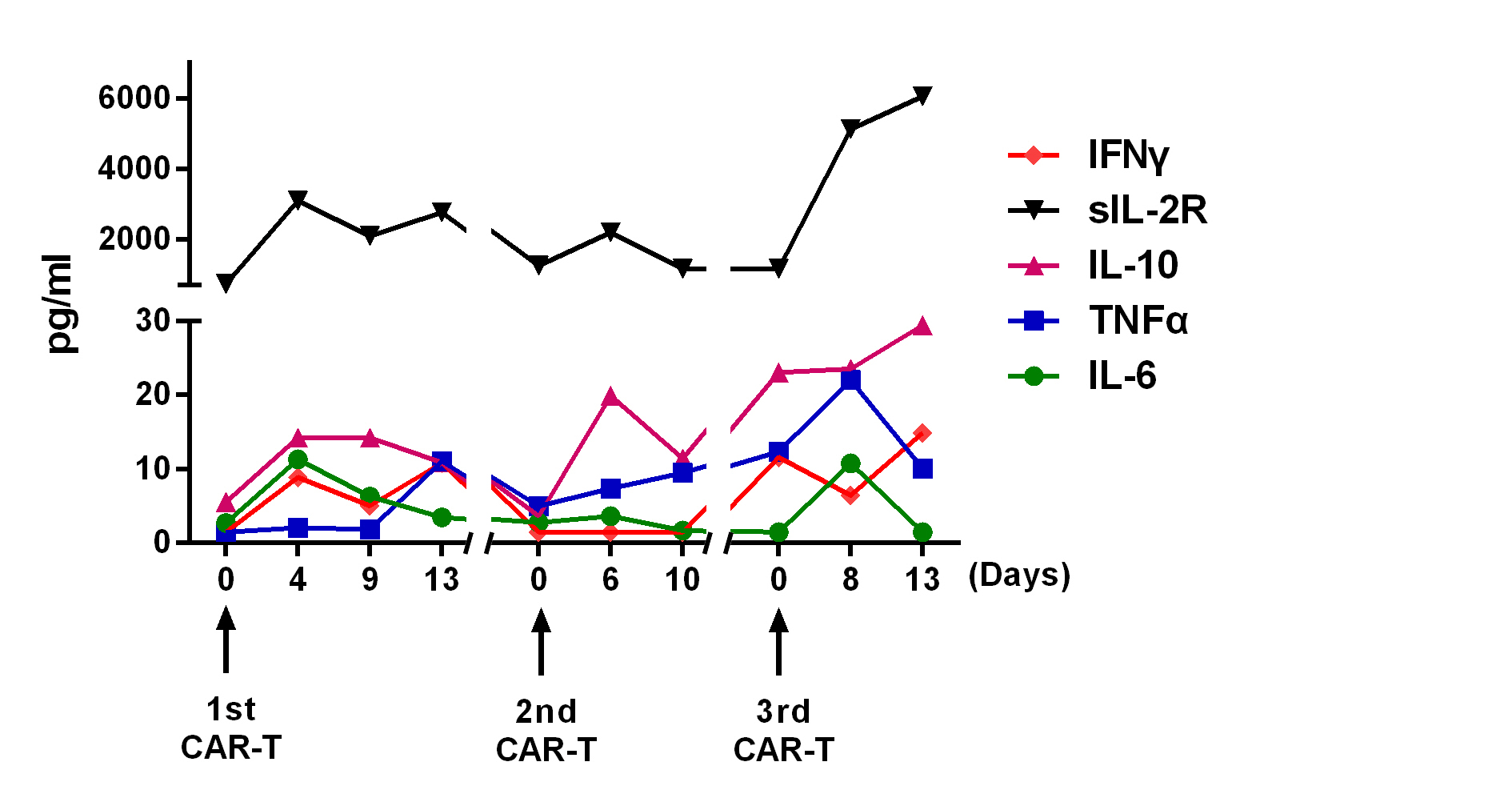


Supplementary Figure 1 Changes of peripheral blood cytokines before and after CAR-T cell therapy


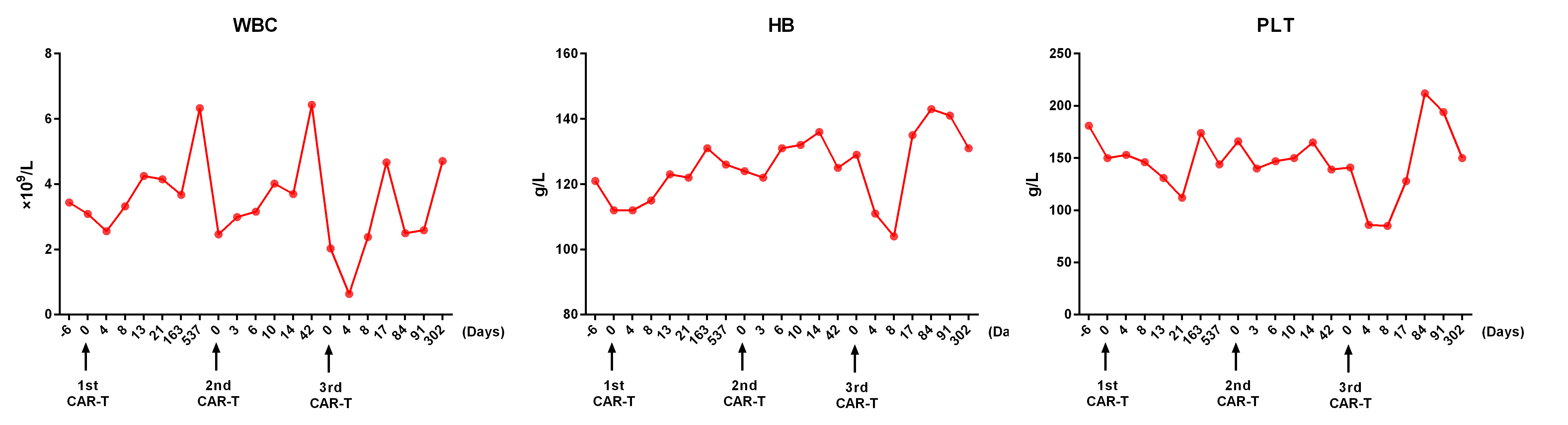


Supplementary Figure 2 Changes of peripheral blood cells before and after CAR-T cell therapy
